# Stabilization of Black Locust Flower Extract via Encapsulation Using Alginate and Alginate–Chitosan Microparticles

**DOI:** 10.3390/polym16050688

**Published:** 2024-03-02

**Authors:** Ivana A. Boškov, Ivan M. Savić, Nađa Đ. Grozdanić Stanisavljević, Tatjana D. Kundaković-Vasović, Jelena S. Radović Selgrad, Ivana M. Savić Gajić

**Affiliations:** 1 Faculty of Technology in Leskovac, University of Nis, Bulevar oslobodjenja 124, 16000 Leskovac, Serbiasavicivan@tf.ni.ac.rs (I.M.S.); 2 Institute for Oncology and Radiology of Serbia, Pasterova 14, 11000 Belgrade, Serbia; 3 Department of Pharmacognosy, Faculty of Pharmacy, University of Belgrade, Vojvode Stepe 450, 11221 Belgrade, Serbia; tatjana.kundakovic@pharmacy.bg.ac.rs (T.D.K.-V.); jelena.radovic@pharmacy.bg.ac.rs (J.S.R.S.)

**Keywords:** black locust flower, UHPLC–ESI–MS analysis, release profile, antioxidant activity, cytotoxic activity, anti-*α*-glucosidase activity

## Abstract

Black locust flower extract contains various polyphenols and their glucosides contribute to the potential health benefits. After intake of these bioactive compounds and passage through the gastrointestinal tract, their degradation can occur and lead to a loss of biological activity. To overcome this problem, the bioactive compounds should be protected from environmental conditions. This study aimed to encapsulate the black flower extract in the microparticles based on biodegradable polysaccharides, alginate, and chitosan. In the extract, the total antioxidant content was found to be 3.18 ± 0.01 g gallic acid equivalent per 100 g of dry weight. Also, the presence of lipids (16), phenolics (27), organic acids (4), L-aspartic acid derivative, questinol, gibberellic acid, sterol, and saponins (2) was confirmed using the UHPLC–ESI–MS analysis. In vitro assays showed that the extract has weak anti-*α*-glucosidase activity and moderate antioxidant and cytotoxic activity against the HeLa cell line. The extrusion method with secondary air flow enabled the preparation of microparticles (about 270 μm) encapsulated with extract. An encapsulation efficiency of over 92% was achieved in the alginate and alginate–chitosan microparticles. The swelling study confirmed a lower permeability of alginate–chitosan microparticles compared with alginate microparticles. For both types of microparticles, the release profile of antioxidants in the simulated gastrointestinal fluids at 37 °C followed the Korsmeyer–Peppas model. A lower diffusion coefficient than 0.5 indicated the simple Fick diffusion of antioxidants. The alginate–chitosan microparticles enabled a more sustained release of antioxidants from extract compared to the alginate microparticles. The obtained results indicated an improvement in the antioxidant activity of bioactive compounds from the extract and their protection from degradation in the simulated gastric conditions via encapsulation in the polymer matrixes. Alginate–chitosan showed slightly slower cumulative antioxidant release from microparticles and better antioxidant activity of the extract compared to the alginate system. According to these results, alginate–chitosan microparticles are more suitable for further application in the encapsulation of black locust flower extract. Also, the proposed polymer matrix as a drug delivery system is safe for human use due to its biodegradability and non-toxicity.

## 1. Introduction

Free radicals that represent highly reactive molecules naturally occur in the human body during various metabolic processes. They serve as a defense mechanism against harmful substances. However, when their production becomes excessive and surpasses the body’s antioxidant capacity, it leads to a state known as oxidative stress [1]. Oxidative stress has a negative impact on all biological systems and is responsible for the pathogenesis of various diseases: atherosclerosis [2], diabetes and related complications [3], cancer [4], etc. In recent years, there has been considerable interest in natural antioxidants derived from plant sources, including phenolic acids, flavonoids, and other bioactive molecules [5]. These compounds are valued for their multifaceted effects on health and functionality. In particular, they exhibit antioxidant, antimicrobial, and other bioactive properties, which makes them very attractive compounds with broad applications in the pharmaceutical, cosmetic, and food industries. Unfortunately, these compounds are subject to degradation under the influence of external factors, which limits their application [6]. In addition, simultaneous gastrointestinal digestion leads to a decrease in antioxidant levels and antioxidant activity [7]. Antioxidant compounds often exhibit astringent effects and have a bitter taste that limits their use in oral formulations [8]. An effective approach to preserve the health-beneficial attributes of antioxidants involves their encapsulation within polymer matrices [9]. This strategy not only enhances their stability and bioavailability but also mitigates unpleasant tastes and elevates the overall quality of the final product. Among the existing encapsulation methods, microencapsulation is considered one of the most efficient techniques to improve the delivery of phytochemicals [10]. It ensures their controlled or sustained release while improving their physicochemical properties. Various biopolymer materials are used for the encapsulation of bioactive compounds, among which polysaccharides have attracted the attention of many researchers [11]. Due to its natural origin, availability, non-toxicity, biocompatibility, and thermal and chemical stability, sodium alginate is a suitable polysaccharide for the encapsulation of bioactive compounds, including plant extracts [12].

Alginates are anionic polysaccharides extracted from different types of algae. It is composed of 1–4 linked *α*-L-guluronic (G) and *β*-D-mannuronic acids (M), alternately arranged in homopolymeric (poly-M and poly-G) and mixed blocks (MG). The G/M ratio determines the permeability and swelling properties of alginate gel [13]. The alginate solution can gel in the presence of divalent cations, such as Ca^2+^, Zn^2+^, or Sr^2+^. The hydrogel is formed by selectively binding two adjacent guluronates in poly-G or MG blocks with multivalent cations (e.g., Ca^2+^ ions), forming a compact structure known as an “egg-box“ [14]. Alginate microparticles are characterized by high porosity, which enables easy and fast diffusion of water or other fluids. This phenomenon leads to a decrease in the yield of encapsulated compounds [15]. Due to this reason, the alginate structure is modified using cationic polyelectrolytes (chitosan). Chitosan, a copolymer of *β*-(1-4)-2-acetamido-D-glucose and *β*-(1-4)-2-amino-D-glucose, is a partially deacetylated product of chitin. It is a mucopolysaccharide isolated from the waste shells of crabs and shrimps. Through electrostatic interactions, chitosan forms a strong complex membrane between its amino residues and carboxyl residues of alginate, with which it coats the surface of alginate microparticles [12]. The prepared microparticles exhibit controlled permeability and mucoadhesive properties. They are stable at pH > 3, making them acceptable for application in the gastrointestinal tract [16]. Alginate–chitosan microparticles can be produced using two methods: either by combining a sodium alginate solution with a CaCl_2_ solution containing chitosan or by incubating calcium alginate microparticles in a chitosan solution [17].

The black locust (*Robinia pseudoacacia* L.) is one of the most widespread, woody, deciduous species around the world [18]. The raw parts of black locusts represent a promising source of antioxidants. Only the flower is used in medicine because the other organs of this plant contain high concentrations of robinin, which is toxic to humans [19]. Robinin is a thermally sensitive compound, so the extract can be safe for use after the preparation at higher temperatures. In traditional folk medicine, the black locust flower is employed to address various health concerns [20]. It is used to treat conditions, such as hemoptysis, metrorrhagia (abnormal uterine bleeding), and other gynecological diseases. Additionally, it is believed to alleviate symptoms associated with colds, fever, migraines, skin diseases, and even bleeding in the colon. Pharmacological studies confirm the antimicrobial [21], antioxidant [20], and anticancer activity of black locust flower extract [22]. These properties of the extract can be attributed to rutin, hyperoside, epigallocatechin, ferulic acid, quercetin, and others [23,24]. Also, the presence of acacetin, secundiflorol, mucronulatol, isomucronulatol, and isovestitol flavonoids in all parts of the black locust was confirmed [25].

Due to the presence of different functional groups in the structure (carboxylic, keto, aldehydic, alcoholic, etc.), the bioactive compounds found in black locust flower extract are susceptible to degradation within the gastrointestinal tract following oral administration. Such degradation can lead to a decrease in their pharmacological activity or to the formation of various degradation products that could pose risks to human health. In this study, the encapsulation of the ethanolic extract of black locust flower in alginate and alginate–chitosan microparticles was carried out to retain the antioxidant activity of the extract and prevent the degradation of antioxidants in gastrointestinal fluids.

## 2. Materials and Methods

### 2.1. Chemicals and Reagents

In this study, absolute ethanol (Sani-Hem doo, Novi Bečej, Serbia), Folin–Ciocalteu’s reagent (Carlo Erba Reagents, Val de Reuil, France), calcium chloride dihydrate, methanol, acetic acid (Zorka Pharm, Šabac, Serbia), gallic acid (purity of 97%) (Merck, Darmstadt, Germany), α-glucosidase, 4-nitrophenyl α-D-glucopyranoside (PNP-G), acarbose, 2,2-diphenyl-1-picrylhydrazyl (DPPH), butylhydroxytoluene (BHT), 3-(4,5-dimethylthiazol-2-yl)-2,5-dyphenyl tetrazolium bromide (MTT), phosphate-buffered saline (PBS), dimethyl sulfoxide (DMSO), sodium dodecyl sulfate (SDS), RPMI 1640 nutrient medium, simulated gastric fluid (pH 1.2, SGF), and simulated intestinal fluid (pH 7.4, SIF) without enzyme (Sigma-Aldrich, St. Louis, MO, USA), alginic acid sodium salt (very low viscosity), water (LC-MS grade), acetonitrile (LC-MS grade), formic acid (Thermo Scientific^TM^, Waltham, MA, USA), chitosan (molecular weight from 100,000 to 300,000) (Acros Organics, Thermo Fisher Scientific, Geel, Belgium) were used. Other used chemicals were pro analysis quality.

### 2.2. Plant Material

A dried black locust flower (*Robinia pseudoacacia* L., Fabaceae) was obtained from the Institute for Medicinal Plants Research “Dr. Josif Pančić” (Belgrade, Serbia). Before extraction, the plant material was ground in an electric mill and sieved on a sieve shaker. A fraction of 0.5 mm was used for further analysis. The moisture content of 10.6% (*m*/*m*) was determined after drying the plant material at 105 °C in a laboratory oven until a constant mass was achieved.

### 2.3. Preparation and Characterization of Black Locust Flower Extract

#### 2.3.1. Extraction Procedure

Black locust flower extract was prepared using ultrasound-assisted extraction in an ultrasonic bath (Sonic, Niš, Serbia). The total power of the device was 3 × 50 W, while the frequency was 40 kHz. The extraction was carried out using 60% (*v*/*v*) ethanol at the extraction temperature of 60 °C and the liquid-to-solid ratio of 10 mL/g for 30 min. The liquid extract was separated from the solid matrix via vacuum filtration.

#### 2.3.2. Total Antioxidant Content

The total antioxidant content (TAC) in the obtained extract was determined according to the spectrophotometric method using Folin–Ciocalteu’s reagent [24]. Its content was expressed as a gram equivalent of gallic acid per 100 g of dry weight (g GAE/100 g d.w.).

#### 2.3.3. Antioxidant Activity According to DPPH Assay

The antioxidant activity of the extract was determined using the DPPH assay. The inhibition of DPPH radicals (*I_DPPH_*) was calculated according to Equation (1):

(1) $$I_{DPPH}\;\left(\%\right)=\frac{A_{c}-\left(A_{s}-A_{b}\right)}{A_{c}\;}\times 100$$

where *A_c_*, *A_s_*, and *A_b_* are the absorbances of the control solution, sample solution, and blank solution at 517 nm, respectively. The synthetic antioxidant BHT was used as a positive control solution. The half maximum inhibitory concentration (IC_50_) was obtained via interpolation from the functionality between the inhibition of DPPH radicals and the sample concentration [24].

#### 2.3.4. Anti-α-Glucosidase Activity

The anti-*α*-glucosidase activity of black locust flower extract was determined using the 400 mU/mL of *α*-glucosidase enzyme solution in a 0.1 M phosphate buffer (pH 6.8). Initially, the stock of the analyzed extract was dissolved in DMSO and then diluted using 0.1 M phosphate buffer (pH 6.8), so the final concentrations were 333.33, 166.67, 83.33, 41.67, 20.83, 10.42, and 5.21 µg/mL. In the 96-well plates, 50 µL of the extract dilutions were preincubated with 50 µL of enzyme solution for each well at 37 °C for 15 min. Afterwards, 50 µL of the substrate solution, 4-nitrophenyl *α*-D-glucopyranoside (1.5 mg/mL PNP-G in phosphate buffer), was added and absorbance A_1_ was measured at 405 nm. This solution was incubated at 37 °C for another 15 min whereupon second absorbance *A*_2_ was measured at 405 nm. Acarbose was used as a positive control. The percentage of enzyme inhibition (*I_e_*) was calculated according to the following formula (Equation (2)):

(2) $$I_{e}\;\left(\%\right)=\frac{A_{2s}-A_{1s}}{A_{2b}-A_{1b}\;}\times 100$$

where *A*_1*b*_, *A*_2*b*_ and *A*_1*s*_, *A*_2*s*_ are the absorbances of the blanks (phosphate buffer, DMSO, enzyme solution, and substrate PNP-G) and sample, respectively. The IC_30_ value (estimated concentration of compounds that caused 30% inhibition of *α*-glucosidase activity) was determined using linear regression analysis [26].

#### 2.3.5. Cytotoxic Activity According to MTT Assay

MTT assay was used to estimate the viability, proliferation, and cytotoxicity of the black locust flower extract. The stock solution was prepared by dissolving the extract in DMSO to the concentration of 400 μg/mL. A solution series (12.5–200 μg/mL) was prepared via dilution of the stock solution in the RPMI 1640 nutrient medium supplemented with 10% fetal bovine serum, 3 mM L-glutamine, 1% penicillin-streptomycin, and 25 mM HEPES buffer and adjusted to pH 7.2 using bicarbonate solution in 96-well microtiter plates (Nunc, Nalgene, Denmark). The HeLa cells (human cervical adenocarcinoma), LS-174 cells (cells of human colon carcinoma), A549 (non-small cell lung carcinoma), and normal MRC-5 cells (human lung fibroblasts) from American-Type Culture Collection (ATCC, Manassas, VA, USA) were seeded in a nutrient medium at 37 °C in a humidified atmosphere (95% air, 5% CO_2_) and incubated for 72 h. The density of HeLa, LS-174, MRC-5, and A549 cells was 2000, 7000, 5000, and 5000 cells/well, respectively. Yellow MTT reagent (20 μL) at the concentration of 5 mg/mL PBS (phosphate buffered saline) was added into each well and incubated for 4 h. After that, the colourimetric reaction occurred and a purple formazan product was formed due to the reduction of MTT dye. Formazan was extracted by adding 100 µL of 10% (*m*/*v*) SDS into the wells. On the next day, the absorbance of the sample was measured at 570 nm on a Multiskan EX reader (Thermo Labsystems, Beverly, MA, USA). A blank solution contained a nutrient medium. The antiproliferative effect of the extract was monitored in relation to the control culture of cells.

The cell’s survival was calculated according to Equation (3) [27]:

(3) $$S\left(\%\right)=\frac{A_{t}-A_{b}}{A_{c}-A_{b}}\times 100$$

where *A_t_*, *A_b_*, and *A_c_* are the absorbances of treated cells with the extract, blank solution, and control solution at 570 nm, respectively. The IC_50_ value represented the sample concentration that inhibited 50% of the cell’s proliferation relative to the untreated control.

#### 2.3.6. Chromatographic Analysis

Validated ultra-high-performance liquid chromatography coupled with mass detection (UHPLC–MS) was used to identify bioactive compounds of black locust flower according to the previously described method with slight modification [28]. The Dionex Ultimate 3000 UHPLC+ system was equipped with a quaternary pump and diode-array detector (DAD). The LCQ Fleet Ion Trap spectrometer (Thermo Fisher Scientific, San Jose, CA, USA) had a heated electrospray ionization probe installed (HESI-II, ThermoFisher Scientific, Bremen, Germany). Xcalibur (version 2.2 SP1.48) and LCQ Fleet (version 2.1) software were used for data acquisition, collection, and analysis. The separation of bioactive compounds was achieved using a Hypersil gold C18 (50 mm × 2.1 mm, 1.9 µm) column at 40 °C. A mobile phase consisted of phase A (water + 0.1% (*v*/*v*) formic acid) and phase B (acetonitrile + 0.1% (*v*/*v*) formic acid). Gradient elution was used as follows: 0–10 min (5–95% B), 10–12 min (95% B), 12–12.1 min (95–5% B), and 12.1–15 min (5% B). The flow rate of the mobile phase was 0.3 mL/min, while the injection volume was 3 µL. The wavelengths of 280, 320, 340, and 360 nm were chosen for spectrum scanning of bioactive compounds. The negative ion mode mass spectra were obtained via full range acquisition from *m*/*z* 110 to 2000 using HESI with the following parameters: source voltage, 5 kV; capillary voltage, −40 V; tube lens voltage, −80 V; capillary temperature, 275 °C; and sheath and auxiliary gas flow (N_2_), 42 and 11 (arbitrary units). A data-dependent scan based on collision-induced dissociation (CID) was used to fragment ions. The normalized collision energy of CID was 35 eV. The bioactive compounds were identified via comparison of the mass of molecular and fragment ions with the available literature data.

### 2.4. Encapsulation of Black Locust Flower Extract in the Microparticles

The alginate microparticles were encapsulated with black locust flower extract using the coaxial air flow extrusion method. An aqueous solution of alginate (1.5%, *m*/*v*) was prepared by mixing alginate overnight to completely dissolve it. Black locust flower extract was added to the alginate solution. The obtained homogeneous solution was transferred to a plastic syringe of 100 mL and added dropwise through a metal needle with a straight-cut tip and a diameter of 26 G (0.45 × 12 mm). The volumetric flow rate of the prepared solution was 33.3 mL/h, while a coaxial air flow pressure was 0.8 bar. The formed drops broke off from the tip of the needle in the form of a stream of tiny droplets under the influence of gravity and coaxial airflow. The spherical microparticles were dropped and solidified in the crosslinking solution of calcium chloride (2%, *w*/*v*) to a total volume of 200 mL. The volumetric ratio of the alginate solution to the calcium chloride solution was 1:2. According to the same procedure, alginate–chitosan microparticles encapsulated with black locust flower extract were prepared. The chitosan solution (0.5%, *m*/*v*) was dissolved in 0.5% (*v*/*v*) acetic acid (acidity regulator).

### 2.5. Characterization of Microparticles

The prepared alginate and alginate–chitosan microparticles encapsulated with the extract were dried before further analysis. The drying process was carried out in a laboratory oven heated to 50 °C for 24 h.

#### 2.5.1. Determination of the Shape and Size of Microparticles

The shape and size of the microparticles were determined using an optical microscope (Leica DM 750, Leica Microsystems, Wetzlar, Germany) equipped with a digital camera. The sphericity factor (*SF*) used to evaluate the roundness of the formed microparticles was calculated according to Equation (4) [29]:

(4) $$SF=\frac{D_{max}-D_{per}}{D_{max}+D_{per}}$$

where *D_max_* is a maximum diameter (mm) passing through the central part of the microparticle; *D_per_* is a diameter directed at *D_max_* that passes through the central part of the microparticle (mm).

Microparticles are ideal spheres if *SF* is around zero. A greater degree of distortion of the shape of the microparticle is achieved when *SF* has higher values.

#### 2.5.2. Determination of Encapsulation Efficiency

Fresh microparticles were vortexed with sodium citrate in a mass ratio of 1:5 for 15 min to destroy their structure. The TAC in the citrate solutions was determined according to the previously described procedure in Section 2.3.2. The encapsulation efficiency of antioxidants was calculated as the ratio of TAC in the citrate solution of destroyed microparticles to the initial extract of black locust flower used for encapsulation.

#### 2.5.3. Swelling Study

The swelling ability of encapsulated alginate and alginate–chitosan microparticles was evaluated gravimetrically under the conditions of SGF and SIF. The swelling of microparticles was analyzed at 37 °C as follows: 0.05 g of dry microparticles were weighed and immersed in 20 mL of the SIF for 2 h, after which the microparticle samples were transferred to 20 mL of the SGF for the next 22 h. Measurements of the mass of the swollen microparticles were performed on an analytical balance. The swelling degree (*SD*) of microparticles was calculated according to Equation (5) [30]:

(5) $$SD\left(\%\right)=\frac{m_{t}-m_{i}}{m_{i}}\times 100$$

where *m_t_* is the mass of swollen microparticles at time *t* and *m_i_* is the mass of dry microparticles (xerogel).

### 2.6. In Vitro Release of Antioxidants from Microparticles

Antioxidant release from dry encapsulated microparticles was monitored under SGF and SIF conditions at 37 °C. About 0.6 g of microparticles were immersed and stirred in 30 mL of SGF at 100 rpm and 37 °C. At certain time intervals, 2 mL of aliquots were taken from the medium. Instead of the aliquot, the equivalent volume of fresh SGF was added to the analyzed solution. The release of antioxidants in SGF was monitored for up to 2 h. After that, the microparticles were filtered, dried, and then combined with 30 mL of SIF preheated to 37 °C with constant stirring at the same temperature. The sampling procedure was repeated according to the previously described method until the microparticles were completely disintegrated. In the collected aliquots (2 mL), the TAC was determined. The obtained results were expressed as the cumulative antioxidant release from microparticles through time. The various kinetic models, including zero order, first order, Higuchi, Hixson–Crowell, Korsmeyer–Peppas, and Baker–Lonsdale models were applied to describe the antioxidant release process from the polymer matrix. Excel add-in DD Solver was used to model the obtained data.

### 2.7. Determination of Antioxidant Activity of the Extract after Its Release from Microparticles in Gastrointestinal Fluids

The antioxidant activity of the extract was also determined after the release of antioxidants from alginate and alginate–chitosan microparticles in SGF and SIF at 37 °C. About 0.1 g of dry microparticles encapsulated with the extract were immersed in 10 mL of SGF or SIF. After 2 h of antioxidants release, 100 μL of the sample was taken and the antioxidant activity was determined.

### 2.8. Statistical Analysis

All data were carried out in triplicate and expressed to be mean ± standard deviation (SD). Analysis of variance (ANOVA) according to multiple range tests was used to determine statistical differences among samples. IBM SPSS Statistics software (version 27, Chicago, IL, USA) was applied to evaluate the statistical differences among samples. The statistically significant differences were those whose *p* < 0.05.

## 3. Results and Discussion

### 3.1. Characterization of Black Locust Flower Extract

#### 3.1.1. Total Antioxidant Content

Before the encapsulation of the black locust flower extract, the TAC of 3.18 ± 0.01 g GAE/100 g d.w. was determined according to the spectrophotometric method. Hallmann [31] reported a tenfold lower TAC of about 3.91 mg/g d.w. for the 80% (*v*/*v*) methanolic extract obtained via maceration with stirring. Unlike this extract, the lyophilized 70% (*v*/*v*) ethanolic extract had almost tenfold higher TAC of 311.93 ± 0.01 mg GAE/g d.w. according to Jurca et al. [32]. These data indicate that the TAC depends on the extraction technique and solvent used. Bratu et al. [33] also determined a TAC of 0.72 ± 0.02 mg GAE/mL for the 50% (*v*/*v*) ethanolic extract obtained via maceration at room temperature for 72 h. This data is not comparable since the unit for the TAC is not expressed as in this study.

#### 3.1.2. Anti-α-Glucosidase Activity

The black locust flower extract showed weak anti-*α*-glucosidase activity. Its IC_30_ value of 291.24 ± 21.23 μg/mL was less than the standard substance, acarbose (IC_50_ of 119.72 ± 4.64 μg/mL). Unlike the weak anti-α-glucosidase activity of the black locust flower extract, the prepared extracts in other similar studies did not show significant activity. The 40% (*v*/*v*) ethanolic extract of flowers from Ganghwa island (Republic of Korea) expressed weaker α-glucosidase inhibitory activity compared to our extract, with an IC_50_ value of 2.39 mg/mL [34]. According to Sarikurkcu et al. [35], among the black locust flower extracts from Turkey, ethyl acetate extract showed the highest anti-α-glucosidase activity (109.01 mg acarbose equivalent/g extract) compared to acetone, methanolic, and aqueous extracts (51.69, 36.76, and 44.68 mg acarbose equivalent/g extract, respectively). These results were correlated with a significant amount of total polyphenols found in the extract: 46.9 mg gallic acid equivalent/g extract, 51.99 mg rutin equivalent/g extract were flavonoids, and even 3.96 mg catechin equivalent/g extract were condensed tannin [35]. So far, it has been shown that polyphenols participate in glucose absorption through α-amylase and α-glucosidase inhibition, which are key enzymes in the digestion of carbohydrates [36]. More precisely, due to their ability to precipitate proteins, hydrolysable and condensed tannins are good enzyme inhibitors. Tannins, especially condensed tannins, are effective inhibitors of α-glucosidase, and their effectiveness can be compared with synthetic inhibitors (acarbose), while the inhibition of α-amylase is mediated by hydrolyzing tannins [37]. On the other hand, the black locust leaf from Japan showed better anti-α-glucosidase activity compared to the flower, where at 50 µg/mL the inhibitory activity was only 15% and at 200 µg/mL activity reached 93%, which was far more than the standard, acarbose (IC_50_ of 13 mg/mL) [38].

#### 3.1.3. Cytotoxic Activity

The cytotoxic activity of the prepared extract was estimated based on an MTT assay. In Table 1, the IC_50_ values of the extract against tumor and healthy cell lines expressed as micrograms per milliliter are depicted. As can be noticed, the extract was only efficient against the HeLa cell line, while in all other cases these values were higher than 200 µg/mL. The compounds were classified based on the IC_50_ values. According to the National Cancer Institute (NCI) of the United States, the extract had moderate cytotoxic activity since the IC_50_ value ranged between 21 and 200 μg/mL [39]. This extract was presented as a tumor-selective agent because it had a weak cytotoxic activity against the MRC-5 cell line (IC_50_ > 200 µg/mL). Uzelac et al. [40] also analyzed the cytotoxic activity of the extract of black locust flower from the territory of Istria (Croatia) during vegetation in 2021. They concluded that the 70% (*v*/*v*) methanolic and 80% (*v*/*v*) ethanolic extracts did not show cytotoxic activity against Vero African green monkey kidney cells because the IC_50_ values (in that paper, IC_50_ is presented as the LC_50_ value) were higher than 1 mg/mL. Bratu et al. [33] also confirmed the cytotoxic activity of 50% (*v*/*v*) ethanolic extract of black locust flower prepared via maceration against HeLa cells. That extract did not express the cytotoxic effect against the palatal mesenchymal stem cells. Cvetković et al. [41] analyzed the cytotoxic activity of methanolic extract of black locust flower obtained using Soxhlet extraction. They concluded that the extract had no significant cytotoxic activity on MRC-5 and MDA-MB-231 (human breast cancer) cell lines. Also, the results of the analysis indicated a great anti-invasive potential in MDA-MB-231 cells.

#### 3.1.4. UHPLC–MS Analysis

**Figure 1:**
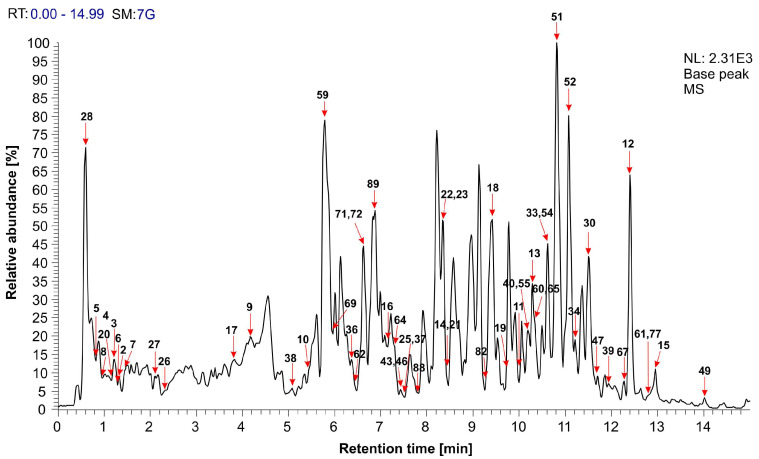
The UHPLC–MS chromatogram of the black locust flower extract (*x*-axis = retention time in minutes; *y*-axis = relative abundance of negative ions in percentages).

The qualitative content of black locust flower extract was determined using the UHPLC–MS method. A base peak chromatogram of the extracts recorded in the negative mode is presented in Figure 1.

The molecular or adduct ions and their fragment ions for 89 quantified compounds, of which 59 are identified compounds, are depicted in Table 2. Among the identified compounds are lipids (16), phenolics (27), organic acids (4), L-aspartic acid derivative, questinol, gibberellic acid, sterol, and saponins (2).

Lipids. Polar lipids (2), phospholipids (6), glycerolipids (1), and fatty acids (7) were detected in the extract. Of the polar lipids, monoacylglycerol in conjugation with sulfoquinovosyl moiety had an [M−H]^−^ ion at *m*/*z* 577 (**55**); i.e., its dimer had an [M−H]^−^ ion at *m*/*z* 1155 (**89**). Compounds **30**, **33**, **34**, **40**, **54**, and **60** were identified as phospholipids with an [M−H]^−^ ion at *m*/*z* 409.4, 431.42, 433.45, 476.41, 571.46, and 595.5, respectively. Among the glycerolipids, monoacylglyceryl glucuronides (C:N 16:0) (**47**) were detected at t_R_ 11.72 min with an [M−H]^−^ ion at *m*/*z* 505.43. Fatty acids represent the most abundant compounds among lipids. Hexadecanedioic acid, which represents a long-chain fatty acid, was assigned as compound **13** with an [M−H]^−^ ion at *m*/*z* 285.39 and t_R_ 10.29 min. The peak at t_R_ 10.01 min originated from oxidized fatty acid (**11**), which had an [M−H]^−^ ion at *m*/*z* 265.28. Compounds with [M−H]^−^ ions at 311.45 (**18**) and 321.06 (**20**) were identified as hydroxy fatty acids. Dihydroxy (**19**) and trihydroxy (**23**) octadecenoic acids had [M−H]^−^ ions at *m*/*z* 313.39 and *m*/*z* 329.43, respectively. Another trihydroxy fatty acid (**21**) with an [M−H]^−^ ion at *m*/*z* 327.47 occurred at t_R_ 8.44 min. Tian et al. [63] also confirmed the presence of various fatty acids in the extract of black locust flower from China.

Phenolics. In the extract, the main polyphenolic classes identified were flavonoids (flavanonols (5), flavonols (7), flavones (3), isoflavones (1), flavanones (1), flavanols (1), anthocyanins (1)), hydroxycinnamic acids (2), phenolic acids (2), phenolic aldehydes (3), and diarylheptanoids (1). Fustin (**14**) with an [M−H]^−^ ion at *m*/*z* 287.47 and its three various derivatives (**49**, **52**, **77**) were from the flavanonol class. A strong antioxidant pinobanksin (**12**), which had the characteristic fragment ions at *m*/*z* 253 and 225, also belongs to this class of compounds. Quercetin, kaempferol, and isorhamnetin as flavonols were noticed in the glycosidic form. Compounds **16** ([M−H]^−^ ion at *m*/*z* 301.35) and **39** ([M−H]^−^ ion at *m*/*z* 463.21) were assigned as quercetin and its glycosidic derivative, respectively. Kaempferol-3-*O*-glucoside (**36**), kaempferol rutinoside (**59**), kaempferol-3-*O*-robinoside-7-*O*-rhamnoside (robinin) (**69**), and kaempferol-3-*O*-(4-coumaroyl)-(feruloyl)-glucoside (isomer) (**71**) had molecular ions at *m*/*z* 447.55, 593.44, 739.41, and 767.46, respectively. The peak at t_R_ 6.44 min with an [M−H]^−^ ion at *m*/*z* 623.06 originated from isorhamnetin-*O*-rutinoside (**62**). The three various flavones (**64**, **67,** and **72**) were identified in the extract, with two being acacetin derivatives (**64** and **72**). Compound **64** quantified at t_R_ 7.34 min gave an adduct ion with formic acid [M−H + HCOOH]^−^ at *m*/*z* 637.43. The effect of collision energy caused the loss of a rhamnoglucoside moiety from this molecule resulting in the appearance of a fragment ion at *m*/*z* 283. Consequently, compound **64** was tentatively assigned to be an acacetin-rhamnoglucoside isomer. The molecular ion at *m*/*z* 767.52 was noted as an acacetin derivative (**72**). According to the literature data, the peak at t_R_ 12.24 min that resulted from diosmetin-7-*O*-glucuronide-3′-*O*-pentoside (**67**) had an [M−H + HCOOH]^−^ ion at *m*/*z* 653.25 and fragment ions at *m*/*z* 607 and 311. Compound **43** ([M−H + HCOOH]^−^ ion at *m*/*z* 491.36) was characterized as isoflavone glycitein-*O*-hexoside. Compound **46** exhibited a molecular ion at *m*/*z* 503.36. This compound corresponds to a liquiritigenin derivative. Flavanol gallocatechin (**17**, [M−H]^−^ at *m*/*z* 305.01) produced fragment ions at *m*/*z* 261, corresponding to the loss of CO_2_ (−44 Da). Among flavonoids, cyanidin derivative (**51**) was also identified based on an [M−H]^−^ ion at *m*/*z* 555.52 and classified as anthocyanin. Ferulic acid (**8**), which belongs to hydroxycinnamic acids, had a peak at t_R_ 0.98 min. Its mass spectrum had a molecular ion at *m*/*z* 239.04. The glycosidic derivative of caffeic acid with an [M−H]^−^ ion at *m*/*z* 387.2 was also found in the chromatogram at t_R_ 0.61 min. Two different peaks with almost the same molecular ions at about *m*/*z* 355 and fragment ions at *m*/*z* 191 (100%) were noted as coumaroylglucaric acid isomers (**26** and **27**). Compounds **2**, **3,** and **7** were identified as phenolic aldehydes, wherein compounds **2** and **3** had approximately the same [M−H]^−^ ions at about *m*/*z* 137 and a main fragment ion at *m*/*z* 91. Compound **7** was the third identified phenolic aldehyde with an adduct ion [M−H + HCOOH + HOH]^−^ at *m*/*z* 224.96 and fragmentation ions at *m*/*z* 179, 161, 143, 119, and 89. This compound was characterized as 3- or 4-hydroxy-2-oxoglutaric acid. A relatively small class of secondary metabolites (diarylheptanoid) belonging to the phenolics group was also identified. Compound **22** (hirsutenone) exhibited an [M−H]^−^ ion at *m*/*z* 327.5 and fragmentation ions at *m*/*z* 309, 291, 239, 197, and 171. Due to their potential therapeutic and organoleptic features, diarylhepatanoids can be considered nutraceuticals.

The identified phenolic compounds in the black locust flower extract are in accordance with previously reported data [40]. The phenolic content of the extract was studied for the black locust flower from different areas of cultivation. Uzelac et al. [40] and Tian et al. [63] analyzed the phenolic content of 70% (*v*/*v*) ethanolic extracts of black locust flowers from the territory of the Istra region (Croatia) and China, respectively. Via mutual comparison of their results, as well as the results obtained in this study, it can be concluded that the climate conditions of plant cultivation and extraction conditions are of crucial significance to the phenolic content. It is known that these phenolic compounds generally have a beneficial effect on human health.

Organic acids. Compound **4** ([M−H]^−^ ion at *m*/*z* 161.07) was assigned as hydroxy oxoglutaric acid (keto acid). The molecular ions at *m*/*z* 191.13, 218.13, and 252.12 (**5**, **6**, and **10**) belong to the organic acids which are used as vitamins.

Amino acids. L-aspartic acid derivative (compound **9**) had an [M−H]^−^ ion at *m*/*z* 250.08.

Quinones. Questinol (compound **15**) with an [M−H]^−^ ion at *m*/*z* 299.36 was noticed at t_R_ 12.94 min.

Hormones. Compound **25** was identified as gibberellic acid with an [M−H]^−^ ion at *m*/*z* 345.58.

Sterols. Hexose conjugate of sterol (compound **38**) with an [M−H]^−^ ion at *m*/*z* 459.23 has occurred at t_R_ 5.1 min.

Glucans. The peak of galacturonoglucan (compound **65**) with an [M−H]^−^ ion at *m*/*z* 647.28 was found at t_R_ 10.38 min.

Saponins. The peaks at t_R_ 8.91 min and t_R_ 7.78 min with an [M−H]^−^ ion at *m*/*z* 957.36 (compound **82**) and an [M−H + HCOOH]^−^ ion at *m*/*z* 1003.37 (compound **88**) were due to the presence of triterpene saponins. Compound **83** (t_R_ = 9.29) had an [M−H]^−^ ion at *m*/*z* 970.98 and fragment ions at *m*/*z* 924, 827, and 719. According to the literature data, the detected compound is most likely saponin [64].

#### 3.1.5. Antioxidant Activity

The antioxidant activity of the extract was estimated based on its IC_50_ value which was found to be 120.9 ± 0.08 µg/mL. The synthetic antioxidant BHT had the IC_50_ value of 35.31 ± 0.12 µg/mL. Antioxidants can be characterized based on their IC_50_ value according to the following classification: “very strong” if the IC_50_ value is less than 50 µg/mL, “strong” if the IC_50_ value is between 50 µg/mL and 100 µg/mL, “moderate” if the IC_50_ value is between 100 µg/mL and 150 µg/mL, and “weak“ if the IC_50_ is 150–200 µg/mL [65]. Based on this classification, the black locust flower extract can be considered a moderate antioxidant, while BHT belongs to the group of very strong antioxidants. Although BHT has better antioxidant activity, the extract is a source of natural antioxidants that, due to its origin, may be more suitable for human health. In the literature, the antioxidant activity of black locust flower is also described regardless of the used solvent and extraction technique. The comparison of obtained data is very hard because of the different units used. According to the DPPH assay, the lyophilized 70% (*v*/*v*) ethanolic extract of black locust flower had an antioxidant capacity of 3.58 ± 0.11% [32]. Bratu et al. [33] determined the antioxidant activity of ethanolic extract to be 0.141 ± 0.02 μM Trolox equivalent/mL using DPPH assay.

### 3.2. Characterization of Microparticles

#### 3.2.1. Shape and Size of Microparticles

**Figure 2:**
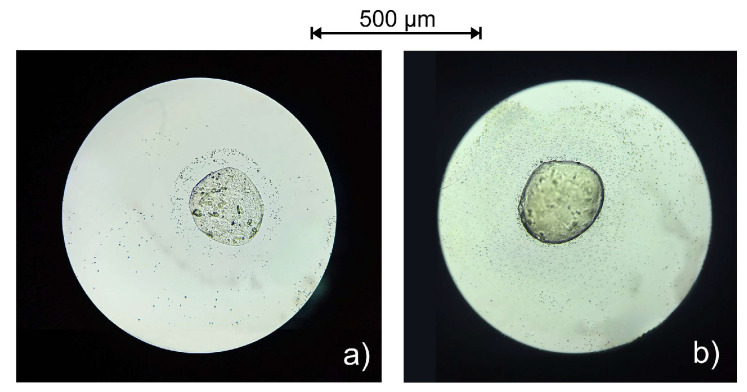
The optical microscopic views of alginate microparticles (**a**) and alginate–chitosan microparticles (**b**) with black locust flower extract at the magnification of 10×.

The size, texture, and shape of encapsulated microparticles, as well as proof of chitosan binding, were estimated based on microscopic analysis. The extract was homogenously distributed within the alginate part of the microparticles. The average particle size of alginate and alginate–chitosan microparticles was 228.0 ± 8.5 and 273.0 ± 10.0 µm, respectively (Figure 2). The increase in microparticle size can be justified by forming a polyelectrolytic membrane as a result of electrostatic interactions between alginate and chitosan. Yousefi et al. [66] also noticed an increase in the size of the alginate–chitosan microparticle encapsulated with *Viola odorata* Linn. extract.

The calculated *SF* values for alginate and alginate–chitosan microparticles were 0.141 ± 0.2 and 0.165 ± 0.3, respectively. These values indicate the absence of an ideal spherical morphology in the majority of prepared microparticles; i.e., the prepared microparticles belong to an elongated shape (*SF* > 0.07). This is in correlation with the other available literature data [67]. The absence of sphericity in the microparticles can lead to a reduction in their mechanical and chemical resistance [68]. It is known that the size and sphericity of alginate–chitosan microparticles, produced via the extrusion dripping method, depend on the process variables (needle size, encapsulation flow surface tension of crosslinking solution, viscosity, and mixing velocity) [68,69]. Having this in mind, the lack of total sphericity for prepared microparticles can be attributed to any mentioned factors. However, since the microparticles are designed for oral administration, the size and shape are not as crucial as those intended for intravenous or intraperitoneal application.

#### 3.2.2. Encapsulation Efficiency

The encapsulation efficiency of black locust flower extract in the alginate and alginate–chitosan microparticles was 92.56 ± 3.21% and 92.05 ± 2.88%, respectively. A significant change in encapsulation efficiency has not occurred due to the presence of the chitosan membrane. The remaining amount of extract was not encapsulated because of its loss and the rapid degradation of unstable compounds during the encapsulation process. Villate et al. [67] also obtained a similar encapsulation efficiency.

#### 3.2.3. Swelling Study

**Figure 3:**
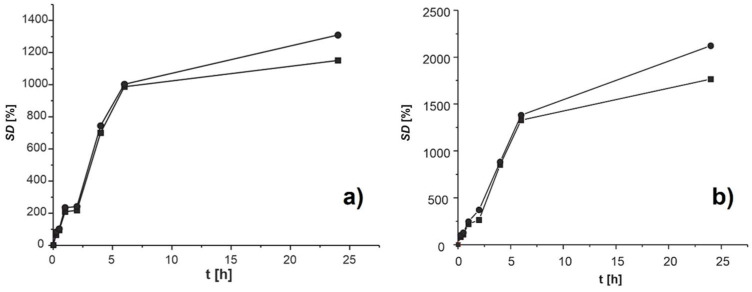
The dependence of swelling degree (*SD*) of empty (■) and encapsulated (●) microparticles of) alginate-chitosan (**a**) and alginate (**b**) on time in SGF and SIF at 37 °C.

One of the most important features of hydrophilic microparticles, such as alginate and alginate–chitosan microparticles, is swelling when coming into contact with water or other fluids at physiological pH values. Kanokpanont et al. [17] reported the swelling ability to be even over 2000% or 20 g of water per 1 g of alginate–chitosan xerogel. The *SD* of microparticles depends on the pH value of the solution [70]. This factor has a significant influence on the release mechanism of encapsulated compounds from the extract. The swelling ability of encapsulated alginate and alginate–chitosan microparticles was monitored in conditions of different pH values at 37 °C for 24 h. Firstly, the microparticles were stored in SGF for 2 h; after that, they were filtered, transferred, and stored in SIF for the next 22 h. The dependences of the *SD* of encapsulated microparticles on time for pH 1.2 and pH 7.4 are depicted in Figure 3.

At pH 1.2, the *SD* of alginate microparticles was intensively grown in the first 2 h reaching a maximal value of 372.2 g of water per g of xerogel (372.2%) (Figure 3). This is probably the result of the hydration of hydrophilic groups in alginate molecules. After transferring the microparticles in SIF (pH 7.4), the *SD* continuously increased up to 2051.6 g/g of xerogel. The *SD* of alginate–chitosan microparticles grew to 241.4 g of water per g of xerogel (241.4%) in the first 2 h (Figure 3). This increase probably occurred due to the hydration of the hydrophilic group in alginate and chitosan molecules as well as the protonation of amino groups of chitosan molecules at lower pH values [71]. In that case, repulsive forces are created, which affect the partial separation of the two polymers and the creation of pores that allow easier penetration of water. The *SD* reached 1309.1 g of water per g of xerogel (1309.1%) after transferring microparticles in SIF (pH 7.4) for 24 h. The high *SD* value indicated the possible separation of polymer chains due to the ionization of carboxylic groups without breaking the chemical bonds in the polymeric net. A similar change in *SD* has been noticed for both types of microparticles in SGF and SIF. The *SD* values for alginate microparticles were significantly higher than those for alginate-chitosan microparticles. The chitosan membrane reduces the permeability of alginate microparticles due to the formation of a polyelectrolytic complex between the amino groups of chitosan and the hydrophilic groups of alginate. The formed complex does not allow easy penetration of fluid inside the alginate–chitosan microparticles. Pravilović et al. [72] also showed a lower *SD* of alginate–chitosan microparticles encapsulated with thyme extracts compared to the alginate microparticles. The results of swelling studies indicated that *SD* increased as the pH value of the medium increased. This behavior of microparticles is desirable since they should be resistant to the conditions of the gastric environment (pH 1.2) but also enable the release of the extract compounds in the conditions of the intestinal environment (pH 7.4).

### 3.3. In Vitro Studies of Antioxidants Release in Simulated Gastrointestinal Fluids

**Figure 4:**
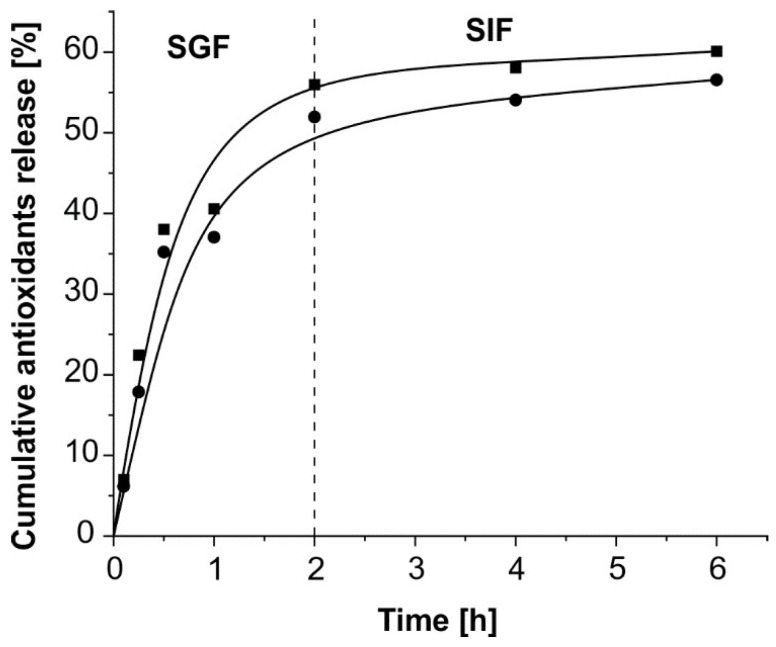
Cumulative antioxidants release (%) from alginate (■) and alginate-chitosan (●) microparticles in simulated gastric fluid (SGF) and simulated intestinal fluid (SIF) at 37 °C.

A cumulative antioxidant release (expressed in percentage) from alginate and alginate–chitosan microparticles under the conditions of SGF and SIF is depicted in Figure 4. Initially, the rapid release of 7.01% and 6.16% of antioxidants from alginate and alginate–chitosan microparticles in SGF occurred within the first 6 min, respectively. This release profile is due to the difference in antioxidant concentrations between the interior and exterior mediums of the microparticles. The sustained release of antioxidants from alginate (55.96%) and alginate–chitosan (51.95%) microparticles was noticed up to 2 h in SGF. The slower release of antioxidants occurred after transferring microparticles in the conditions of SIF. After 6 h in SIF, exactly 60.11% and 56.56% of antioxidants were released from alginate and alginate–chitosan microparticles, respectively. Villate et al. [67] reported an analogous in vitro release profile of cannabinoids from alginate–chitosan microparticles using a similar model. In their study, the cumulative release of cannabinoids in SGF during the first 2 h was significantly lower (about 20% of the total encapsulated compound). They obtained almost the same percentage of cumulative release as in this study after 6 h. The low pH value of SGF impacted the formation of alginic acid in alginate which interfered with antioxidant release from microparticles; i.e., it led to the low cumulative release [73]. The acidic degradation of antioxidants in the gastric environment is one of the factors that negatively impacts their oral bioavailability [74].

In this study, the antioxidants release profiles in both types of microparticles were quite similar. The percentage of released antioxidants from alginate microparticles was higher than from alginate–chitosan microparticles, which was caused by the presence of a chitosan membrane [75]. The membrane reduced the diffusion of antioxidants from the inner parts of microparticles to the exterior environment and extended the drug release profile [76]. The zero order, first order, Higuchi, Hixson–Crowell, Korsmeyer–Peppas, and Baker–Lonsdale models were applied to fit the obtained data for antioxidant release from the polymeric matrix. The fit goodness of the experimental data was estimated based on the statistical parameters, such as the coefficient of determination (*R*^2^), adjusted coefficient of determination (*R_adj_*^2^), root mean square error (*RMSE*), and Akaike information criterion (*AIC*). It is recommended that the model has a higher coefficient of determinations and lower *RMSE* and *AIC*. The Korsmeyer–Peppas model was proved to be the best model for describing both release profiles because the highest *R*^2^ value (around 0.84) and lowest *AIC* value (around 44) were found in this model (Table 3). The *R*^2^ value of 0.84 showed that 84% of the variance in antioxidant release could be explained using this model. The Korsmeyer–Peppas model is suitable for use in drug release from hydrogels or other systems which change shape and volume during this process. In this model, the diffusion coefficients of 0.305 and 0.322 were obtained for antioxidant release from alginate and alginate–chitosan microparticles, respectively. The values of *n* less than 0.5 indicate the simple Fick diffusion. In the case of values greater than 0.5, a non-Fickian release occurs indicating diffusion in the hydrated matrix and relaxation of the polymer. Since the diffusion exponents were lower than 0.5, the release of antioxidants was subjected to the simple Fickian diffusion. This fact implied the controlled release of antioxidants via a diffusion process [77]. Fickian diffusion is defined by a high rate of solvent diffusion into the matrix and a low rate of polymeric relaxation.

### 3.4. Antioxidant Activity of the Extract after Its Release from Microparticles in Gastrointestinal Fluids

The antioxidant activity of black locust flower extract encapsulated in alginate and alginate–chitosan microparticles was determined after 2 h in SGF or SIF. The calculated IC_50_ values of the samples are depicted in Table 4. With both types of microparticles, a higher antioxidant activity of the extract was noticed in the conditions of SIF. The extract encapsulated in alginate–chitosan microparticles expressed a better antioxidant activity compared to the extract encapsulated in alginate microparticles in the conditions of SIF. In this case, the chitosan membrane also had an important role in preventing the loss of antioxidant activity of the extract in the conditions of SGF. The obtained data are expected considering the alginate microparticles are degraded at the higher pH values, while they are stable in an acidic environment. The chitosan membrane enhances the microparticle stability, causing the reduction in antioxidants released in SGF [17]. Comparing the IC_50_ values of the extract before (120.9 ± 0.08 µg/mL) and after encapsulation, it can be noticed that the antioxidant activity significantly increased. The reason for such behavior is probably due to the synergistic effect of the extract, alginate [78], and chitosan [79], which is also known to possess antioxidant activity.

According to the carried-out analyses, the alginate–chitosan microparticles were shown as a more suitable drug delivery system compared to the pure alginate microparticles due to the slower antioxidants release in simulated gastrointestinal fluids and better antioxidant activity of the sample. The main contribution of this study is the preparation of microparticles encapsulated with black locust flower extract at the laboratory level by protecting its antioxidant activity. To provide the scale enlargement of microparticle production, the procedure should be adjusted to the appropriate equipment at the industrial level.

## 4. Conclusions

The ethanolic extract of black locust flowers represents a source of antioxidant compounds with a TAC of 3.18 ± 0.01 g GAE/100 g d.w. The UHPLC–ESI–MS method also indicated the presence of 27 phenolic compounds. According to MTT and DPPH assays, the extract had moderate antioxidant and cytotoxic activity against the HeLa cell line. The extract can be considered a tumor-selective agent due to having no cytotoxic effect against the normal MRC-5 cell line. To protect and stabilize the extract from the conditions of the gastrointestinal tract in vitro, the encapsulation via extrusion method was used for the preparation of microparticles based on alginate and alginate–chitosan. In alginate–chitosan microparticles, the chitosan membrane reduced the permeability of alginate microparticles and directly impacted the protection of antioxidants and their low diffusion from the inner part of microparticles to the exterior medium. Also, the antioxidant activity of the extract was improved after its encapsulation in the microparticles. This is most likely caused by the synergistic effects of chitosan and alginate, which have expressed antioxidant activity. In summary, the prepared microparticles had standard quality characteristics in gastrointestinal environments. The procedure for microparticle preparation did not require the use of expensive equipment and toxic solvents and did not produce any hazardous waste harmful to human health or the environment. All research was carried out at the laboratory level. For further commercial application of these microparticles, it is necessary to study new industrial equipment and procedures for their preparation.

## Figures and Tables

**Table 1:** IC_50_ values (*n* = 3) of black locust flower extract according to the MTT assay.

Cell Lines	IC_50_ (µg/mL)
HeLa	161.99 ± 6.88
MRC-5	>200
LS-174	>200
A549	>200

**Table 2:** The identified compounds in the black locust flower extract using the UHPLC–MS method.

No.	R_t_, min	Molecular or Adduct Ion, *m*/*z*	Fragment Ions, *m*/*z*	Compound	Class of Compounds	References
1	1.41	116.96	89 (100%)	unknown		
2	1.35	136.67	91 (100%)	protocatechuic aldehyde	phenolic aldehyde	[42]
3	1.27	136.97	109, 91 (100%)	2,4-dihydroxybenzaldehyde	phenolic aldehyde	[42]
4	0.95	161.07	143, 115, 99 (100%), 89, 57	3- or 4-hydroxy-2-oxoglutaric acid	keto organic acid	[43]
5	0.81	191.13	173, 145, 129, 111 (100%), 101	citric acid	organic acid	[43]
6	1.29	218.13 ^a^	200, 130, 99, 88 (100%)	D-(+)-pantothenic acid	organic acid (vitamin)	
7	1.44	224.96 [M−H + HCOOH + HOH]^−^	179 (100%), 161, 143, 119, 89	3- or 4-hydroxy-2-oxoglutaric acid	phenolic aldehyde	
8	0.98	239.04 ^a^ [M−H + HCOOH]^−^	193 (100%), 165, 124, 114	ferulic acid	hydroxycinnamic acid	
9	4.13	250.08	204, 146, 132 (100%), 115, 91, 88	L-aspartic acid derivative	α-Amino acid	
10	5.44	252.12 ^a^	234, 137, 136 (100%), 92	4-amino benzoic acid derivative	organic acid	
11	10.01	265.28 ^a^	97 (100%)	oxidized fatty acid	fatty acid	
12	12.4	271.3	253, 225 (100%)	pinobanksin	flavanonol	[44]
13	10.29	285.39	267, 223 (100%)	hexadecanedioic acid	fatty acid	[45]
14	8.41	287.47	269 (100%), 223, 211, 169, 155, 139	fustin	flavanonol	[46]
15	12.94	299.36	253 (100%)	questinol	quinone	[47]
16	7.17	301.35	273, 255, 239, 193, 179 (100%), 151, 107	quercetin	flavonol	[48]
17	4.07	305.01	261 (100%), 224, 201, 181, 128	gallocatechin	flavanol	[49]
18	9.43	311.45	293 (100%), 275, 253, 235, 223, 201, 183, 171	hydroxydioxoheptadecenoic acid	fatty acid	[50]
19	9.75	313.39	295 (100%), 277, 213, 201, 195, 183, 179, 171, 129	dihydroxy-octadecenoic acid	fatty acid	[51]
20	1.18	321.06	277 (100%), 259, 215, 128	12-hydroxy-6, 8, 10, 13-octatetraenedioic acid	fatty acid	[52]
21	8.44	327.47	309 (100%), 291, 263, 251, 225, 209, 197, 183	9,12,13-trihydroxyoctadecadienoic acid	fatty acid	[50]
22	8.33	327.5	309, 291 (100%), 239, 197, 171	hirsutenone	diarylheptanoid	[53]
23	8.3	329.43	311, 293, 229, 211, 171 (100%)	11,12,13-trihydroxyoctadecenoic acid	fatty acid	[50]
24	12	343.41	325, 283 (100%), 254, 225, 211	unknown		
25	7.52	345.58 ^a^	327, 317, 301, 291, 285, 271, 245, 239, 229, 215 (100%), 195, 181	gibberellic acid	plant hormone	
26	2.27	355.04	337, 216, 209, 191 (100%), 173, 129	coumaroylglucaric acid isomer	phenolic acid	[54]
27	2.07	355.27	337, 322, 309, 209, 191 (100%), 173, 147	coumaroylglucaric acid isomer	phenolic acid	[54]
28	0.61	387.2 [M−H + HCOOH]^−^	341 (100%), 251, 195, 179	caffeic acid hexoside	hydroxycinnamic acid	[55]
29	4.73	395.14	377, 349 (100%), 179, 143	unknown		
30	11.47	409.4	361, 251, 171, 153 (100%)	lyso-phosphatidic acid (16:0)	phospholipid	[56]
31	12.86	411.32	368, 281, 147, 129 (100%)	unknown		
32	6.95	423.57	405, 279 (100%), 249, 235, 205, 169, 139, 122	unknown		
33	10.57	431.42	171, 153 (100%)	N-acylglycerophosphatidylethanolamine (18:3)	phospholipid	[56]
34	11.19	433.45	329, 313, 279, 171, 153 (100%)	N-acylglycerophosphatidylethanolamine (18:2)	phospholipid	[56]
35	12.56	437.37	420, 313, 285, 279, 263, 251, 171, 153 (100%)			
36	6.35	447.55 ^a^	357, 327, 285 (100%), 255, 241, 165	kaempferol-3-*O*-glucoside	flavonol	
37	7.55	449.58 ^a^	431, 413, 403, 353, 327, 301, 287 (100%), 269, 251, 239	isookanin-7-*O*-glucoside	flavanone	
38	5.1	459.23	441, 399, 381, 295, 287, 242, 173, 157 (100%)	sterol-hexose conjugates (ST21:3;O;Hex)/sterol (ST 27:4;O6)	sterol	[50]
39	11.93	463.21	445, 426, 417 (100%), 399, 356, 345, 301, 255, 161	quercetin-3-*O*-glucoside	flavonol	[48]
40	10.23	476.41	402, 384, 277, 233, 171, 153 (100%)	N-acylglycerophosphatidylethanolamine (18:2)	phospholipid	[56]
41	12.71	480.46	434, 412, 390, 350, 279, 200 (100%)	unknown		
42	7.4	483.57	465, 439, 421, 391 (100%), 229, 172, 153	unknown		
43	7.43	491.36 [M−H + HCOOH]^−^	445, 343, 303, 283 (100%)	glycitein-o-hexoside	isoflavone	[57]
44	13.52	499.42	313, 261, 255 (100%), 243, 187	unknown		
45	5.65	501.18	483, 403, 250, 206 (100%), 164, 147	unknown		
46	7.37	503.36	459 (100%), 441, 365, 345, 327, 281, 187	liquiritigenin derivative	flavanone	[57]
47	11.72	505.43	487, 467, 361, 267, 255 (100%), 249, 231, 205, 189	monoacylglyceryl glucuronides (16:0)	glycerolipid	[50]
48	6.56	511.47	493, 452, 431 (100%), 285	unknown		
49	14.05	547.51 ^a^	287 (100%), 277, 269	fustin derivative	flavanonol	
50	11.81	555.13	509, 486, 475 (100%), 280	unknown		
51	10.82	555.52 ^a^	538, 495, 476, 390, 285 (100%), 269, 223, 195	cyanidin derivative	anthocyanin	
52	11.16	557.66 ^a^	287 (100%), 269, 239, 221	fustin derivative	flavanonol	
53	10.76	564.37	520, 504 (100%), 279, 251	unknown		
54	10.7	571.46	409, 391, 315, 283, 255 (100%), 241	palmitoyl-glycerophosphoinositol	phospholipid	[58]
55	10.13	577.43	532, 514, 475, 317, 299 (100%), 225, 207, 165	sulfoquinovosyl monoacylglycerols (18:3)	polar lipid	[50]
56	10.07	577.55	521, 469, 371, 299 (100%), 277, 225, 207, 189, 165	unknown		
57	12.37	579.34	445, 410, 392 (100%), 323, 256, 187	unknown		
58	11.9	583.46	537, 442, 299 (100%), 287, 225, 207, 183, 165	unknown		
59	6.22	593.44	549, 339, 327, 285 (100%), 255, 239, 227, 211, 186	kaempferol rutinoside	flavonol	[54]
60	10.32	595.5	507, 415, 341, 315, 279 (100%), 261, 241, 223	lyso-phosphatidylinositols (18:2)	phospholipid	[56]
61	12.82	607.32	575 (100%), 563, 531, 487, 475, 329, 311, 295, 277	diosmetin-7-*O*-glucuronide-3′-*O*-pentoside	flavone	[59]
62	6.44	623.06	577 (100%), 507, 350, 300	isorhamnetin-O-rutinoside	flavonol	[48]
63	11.22	625.28	579 (100%), 557, 341, 306, 287	unknown		
64	7.34	637.43 [M−H + HCOOH]^−^	591 (100%), 335, 283, 268	acacetin-rhamnoglucoside isomer	flavone	[59]
65	10.38	647.28	601 (100%), 485, 323	galacturonoglucan	glucan	[60]
66	11	649.33	621, 603 (100%), 423	unknown		[56]
67	12.24	653.25 [M−H + HCOOH]^−^	607 (100%), 311	diosmetin-7-*O*-glucuronide-3′-*O*-pentoside	flavone	[59]
68	13.14	721.56	683, 678, 595, 465 (100%), 416, 409, 391, 329, 255	unknown		
69	5.94	739.41	593 (100%), 431, 369, 285, 246	kaempferol-3-*O*-robinoside-7-*O*-rhamnoside (robinin)	flavonol	[61]
70	9.51	763.82	632, 613, 571, 551, 525, 498, 455 (100%), 437, 407, 358, 249	unknown		
71	6.69	767.46	749, 707, 657, 483, 325, 283 (100%), 268	kaempferol-3-*O*-(4-coumaroyl)-(feruloyl)-glucoside (isomer)	flavonol	[62]
72	6.66	767.52 ^a^	749, 592, 483, 283 (100%), 268, 240	acacetin derivative	flavone	
73	6.98	769.25	723 (100%), 415, 283	unknown		
74	13.6	774.26	728 (100%), 579	unknown		
75	9.61	793.78	750, 613, 603, 454 (100%), 436	unknown		
76	6.79	799.24	753 (100%), 529	unknown		
77	12.79	827.75	810, 720, 626, 558, 539, 287 (100%), 269	fustin derivative	flavanonol	[46]
78	8.8	881.82	838 (100%), 778, 723, 381	unknown		
79	12.21	883.55	721, 391, 335, 329 (100%), 291	unknown		
80	9.65	921.73	876, 822 (100%), 741, 652, 585, 564, 457, 401	unknown		
81	5.37	947.08	991 (100%), 787	unknown		
82	8.91	957.36	822, 797, 708, 498 (100%), 453	triterpenoid saponin	saponin	
83	9.29	970.98	924 (100%), 827, 719	unidentified saponin	saponin	
84	9.25	971.3	840 (100%), 714	unknown		
85	8.87	985.44	819 (100%), 595, 447	unknown		
86	13.04	997.28	966, 746, 718 (100%)	unknown		
87	8.15	1001.05	992, 982, 934, 363 (100%)	unknown		
88	7.78	1003.37 [M−H + HCOOH]^−^	959, 896 (100%), 640	triterpenoid saponin	saponin	
89	6.85	1155.44	577 (100%)	sulfoquinovosyl monoacylglycerols (18:3) dimer	polar lipid	

Numbers in parentheses (C:N) indicate the number of carbon atoms (C) and double bonds (N) in the fatty acid side chains. R_t_—retention time. ^a^ https://massbank.eu/MassBank/ (available 1 February 2024).

**Table 3:** Kinetic models and statistics parameters of fitting the data of antioxidant release from alginate and alginate–chitosan microparticles at 37 °C.

Kinetic Model	Equation	Alginate Microparticle		Alginate–Chitosan Microparticle	
		Parameter	Goodness of Fit	Parameter	Goodness of Fit
Zero order	$F=k_{0}t$	*k*_0_ = 13.446	*R*^2^ = –0.4241 *R_adj_*^2^ = –0.4241 *RMSE* = 23.7987 *AIC* = 58.9172	*k*_0_ = 12.547	*R*^2^ = –0.2469 *R_adj_*^2^ = –0.2469 *RMSE* = 21.4682 *AIC* = 57.4743
First order	$F=100\times \left(1-e^{-k_{1}t}\right)$	*k*_1_ = 0.299	*R*^2^ = 0.2264 *R_adj_*^2^ = 0.2264 *RMSE* = 17.5400 *AIC* = 54.6451	*k*_1_ = 0.247	*R*^2^ = 0.2719 *R_adj_*^2^ = 0.2719 *RMSE* = 16.4048 *AIC* = 53.7084
Higuchi model	$F=k_{H}\sqrt{t}$	*k_H_* = 30.572	*R*^2^ = 0.6575 *R_adj_*^2^ = 0.6575 *RMSE* = 11.6704 *AIC* = 48.9411	*k_H_* = 28.372	*R*^2^ = 0.7112 *R_adj_*^2^ = 0.7112 *RMSE* = 10.3328 *AIC* = 47.2368
Korsmeyer–Peppas model	$F=k_{KP}t^{n}$	*k_KP_* = 38.296 *n* = 0.305	*R*^2^ = 0.8675 *R_adj_*^2^ = 0.8410 *RMSE* = 7.9514 *AIC* = 44.2930	*k_KP_* = 34.892 *n* = 0.322	*R*^2^ = 0.8701 *R_adj_*^2^ = 0.8442 *RMSE* = 7.5899 *AIC* = 43.6414
Hixson–Crowell model	$F=100\times \left(1-{\left(1-k_{HC}t\right)}^{3}\right)$	*k_HC_* = 0.076	*R*^2^ = 0.0204 *R_adj_*^2^ = 0.0204 *RMSE* = 19.7379 *AIC* = 56.2979	*k_HC_* = 0.065	*R*^2^ = 0.1062 *R_adj_*^2^ = 0.1062 *RMSE* = 18.1759 *AIC* = 55.1437
Baker–Lonsdale model	$\frac{3}{2}\times \left(1-{\left(1-\frac{F}{100}\right)}^{\frac{2}{3}}\;\right)-\frac{F}{100}=k_{BL}t$	*k_BL_* = 0.024	*R*^2^ = 0.7877 *R_adj_*^2^ = 0.7877 *RMSE* = 9.1895 *AIC* = 45.5952	*k_BL_* = 0.019	*R*^2^ = 0.8105 *R_adj_*^2^ = 0.8105 *RMSE* = 8.3700 *AIC* = 44.2875

*R*^2^—coefficient of determination; *R_adj_*^2^—adjusted coefficient of determination; *RMSE*—root mean square error; *AIC*—Akaike information criterion; *F*—cumulative antioxidants release; *k_0_*, *k_1_*, *k_H_*, *k_KP_*, *k_HC_*, *k_BL_*—release constant of zero order, first order, Higuchi, Korsmeyer–Peppas, Hixson–Crowell, Baker–Lonsdale, respectively.

**Table 4:** IC_50_ values (µg/mL) of encapsulated extract of black locust flowers in alginate–chitosan and alginate microparticles in SGF and SIF.

Microparticle	Simulated Gastric Fluid	Simulated Intestinal Fluid
Alginate	109.4 ± 0.06	89.6 ± 0.07
Alginate–chitosan	115.7 ± 0.03	68.3 ± 0.05

## Data Availability

Data are contained within the article.
